# LncRNA PART‐1 targets TGFBR2/Smad3 to regulate cell viability and apoptosis of chondrocytes via acting as miR‐590‐3p sponge in osteoarthritis

**DOI:** 10.1111/jcmm.14690

**Published:** 2019-10-01

**Authors:** Chao Lu, Zheng Li, Shouye Hu, Yuanzhen Cai, Kan Peng

**Affiliations:** ^1^ Department of Joint Surgery Xi'an Honghui Hospital Xi'an Jiaotong University Health Science Center Xi'an China; ^2^ Department of Emergency the First Affiliated Hospital of Xi'an Medical University Xi'an China

**Keywords:** apoptosis, chondrocytes, miR‐590‐3p, osteoarthritis, PART‐1, TGFBR2

## Abstract

Osteoarthritis (OA) is a degenerative joint disease that commonly occurs in the elderly. This study focused on apoptosis and explored the modulating effects of long non‐coding (lncRNAs) prostate androgen‐regulated transcript‐1 (PART‐1) on chondrocytes apoptosis. In the present study, the PART‐1 expression level was down‐regulated in the OA cartilages. Silence of PART‐1 decreased the cell viability and promoted chondrocytes apoptosis. Overexpression of PART‐1 could reverse the effects induced by interleukin 1β (IL‐1β) stimulation, thus slowing down the apoptosis rate. MiR‐590‐3p was found to be the potential target, and RNA immunoprecipitation and luciferase activity assay confirmed the binding between PART‐1 and miR‐590‐3p. Moreover, miR‐590‐3p was down‐regulated by PART‐1 and was negatively associated with PART‐1. Transforming growth factor‐beta receptor type 2 (TGFBR2) was positively associated with PART‐1. Down‐regulation of PART‐1 decreased cell viability and induced cell apoptosis, which was partially reversed by miR‐590‐3p silence or TGFBR2 overexpression; while overexpression of PART‐1 increased the cell viability and decreased the caspase 3 activity and apoptotic rates, and the effects were partially attenuated by miR‐590‐3p overexpression or silence of TGFBR2 in IL‐1β‐stimulated chondrocytes. Knock‐down of PART‐1 down‐regulated both Smad3 and p‐Smad3 protein levels, which was reversed by miR‐590‐3p inhibition or TGFBR2 overexpression. Smad3 expression level was lower in the OA group than that in the normal group and was positively associated with the PART‐1 expression level. Collectively, the study revealed that lncRNA PART‐1 regulates the apoptosis of chondrocytes in OA by acting as a sponge for miR‐590‐3p, which subsequently regulates TGFBR2/Smad3 signalling.

## 1 INTRODUCTION

Osteoarthritis (OA) is a degenerative joint disease that commonly occurs in the elderly. It is reported that around 15% of the population suffer from OA in the world.^1^, ^2^ Based on the results from epidemiological survey in China, about 4% of Chinese population is diagnosed with OA. For the people over 60 years old, the incidence of OA reaches as high as 42.8%.^3^ In the early stages, the treatment of OA focuses on pain relief and symptom control.^4^, ^5^ In the advanced stage, there will be activity disorders and even disability, and joint replacement surgery will be the only therapeutic option. Thus, understanding of the pathology is critical for the development of novel therapeutic drugs. Recent studies showed that apoptosis of chondrocytes has been detected in the OA cartilage and is a key factor for OA progression.^6^ Inhibition of chondrocytes apoptosis may be achieved through genetic or environmental modulation. Understanding the underlying molecular mechanisms of chondrocytes apoptosis is key for us to find novel therapeutic targets for the management of OA.

Non‐coding RNAs (ncRNAs) are a class of RNA molecules that do not have the ability to encode proteins.^7^ Depending on the length of the nucleotides, ncRNAs can be divided into microRNAs (miRNAs) and long non‐coding RNAs (lncRNAs). Studies have shown that miRNAs are abnormally expressed in the OA and affect the integrity of cartilage.^8^ In addition, results from several expression profiling studies have suggested a correlation between lncRNAs and perturbed articular cartilage. LncRNA neuroendocrine secretory protein antisense was elevated in OA and was associated with OA pathogenesis.^9^ Cartilage injury‐related lncRNA promotes chondrocyte extracellular matrix degradation in OA.^10^ In addition, expression level of lncRNA plasmacytoma variant translocation 1 (PVT1) was up‐regulated in OA chondrocytes and silencing PVT1 inhibited the apoptosis of OA chondrocytes, and overexpression of PVT1 promoted the apoptosis of normal chondrocytes.^11^ Recently, Zhao et al presented genome‐wide identification of lncRNAs in patients with intervertebral disc degeneration and spinal cord injury. RNA‐seq profiling indicated that 1854 lncRNAs were differentially expressed, of which, lncRNA prostate androgen‐regulated transcript‐1 (PART‐1) was up‐regulated and was the core element of competing endogenous RNA.^12^ Accordingly, we hypothesize that PART‐1 may play a key role in the development of OA.

In the present study, we explored the biological actions of lncRNA PART‐1 on chondrocytes in OA and uncovered PART‐1 acted as a sponge for miR‐590‐3p, which in turn regulated transforming growth factor‐beta receptor type 2 (TGFBR2)/SMAD family member 3 (Smad3) axis.

## 2 MATERIALS AND METHODS

### 2.1 Human articular cartilage tissues

The OA articular cartilages were obtained from patients subjected to total knee replacement in the Xi'an Honghui Hospital between January 2014 and June 2018. The normal articular cartilages were isolated from patients underwent non‐OA hip replacement surgery or traumatic amputees in the Xi'an Honghui Hospital between June 2014 and June 2018. The study was approved by the Human Ethics Committee of Xi'an Honghui Hospital. Informed consent was signed and obtained from all donors.

### 2.2 Chondrocyte cultures

The immortalized human chondrocytes cell lines C20/A4 (American Type Culture Collection, Manassas, USA) was cultured in DMEM/F12 supplemented with 10% foetal bovine serum (FBS; Thermo Fisher Scientific) and L‐glutamine (Sigma‐Aldrich). The cells were maintained in a humidified atmosphere with 5% CO_2_ at 37°C.

### 2.3 Quantitative real‐time PCR (qRT‐PCR)

Total RNA from tissues or cells were extracted by Trizol reagent (Invitrogen) and reversely transcribed to cDNA. Quantitative analysis of the lncRNA, mRNA or miRNA expression was performed by using SYBR^®^ Premix Ex Taq^™^ kit (Takara) in an ABI7900 system (Applied Biosystems). Glyceraldehyde 3‐phosphate dehydrogenase was used as an internal control for lncRNA and mRNA expression, while U6 was the internal control for miRNA expression.

### 2.4 Oligonucleotides and cell transfection

The coding sequence of PART‐1 and TGFBR2 was subcloned into the pcDNA3.1 (+) vector (Invitrogen). PART‐1 siRNA, TGFBR2 siRNA, nonsense siRNA, miR‐590‐3p mimic, miR‐590‐3p inhibitor and respective controls were purchased from Guangzhou Ribobio company. Chondrocytes were transfected with the above plasmids, siRNAs or miRNAs using Lipofectamine 3000 (Invitrogen), and transfected chondrocytes were collected for in vitro assays at 24‐72 hours after transfection. In some experiments, the cells were stimulated with 5 ng/mL interleukin 1β (IL‐1β; Sigma‐Aldrich) for 24 hours.

### 2.5 Cell counting kit‐8 (CCK‐8) assay

The cell viability was assessed by using CCK‐8 assay kit (Dojindo). Treated chondrocytes were seeded in 96‐well plates at the density of 1000 cells/well. After 24‐72 hours incubation, cells were then added with the CCK‐8 reagent and incubated for 2 hours at room temperature. Then, the optical density values were measured at 450 nm to determine the cell proliferative ability.

### 2.6 Caspase‐3 activity assessment

The caspase‐3 activity was determined by specific caspase‐3 activity kit (R&D Systems) according to the manufacturer's instructions. After transfection, cell lysates were collected and incubated with reaction buffer supplied with caspase substrates for 1 hour in the dark. The caspase‐3 activity was assessed by measuring the absorbance at a wavelength of 450 nm.

### 2.7 Flow cytometry analysis

The cell apoptosis was detected by fluorescein isothiocyanate (FITC)‐Annexin V/propidium iodide (PI) Apoptosis Detection kit (Thermo Fisher Scientific). Briefly, the cells with different treatments were washed with cold phosphate saline buffer and trypsinized, and the cells were then re‐suspended in 100 μL binding buffer containing FITC‐Annexin V/PI. The cells were then incubated at room temperature in the dark for 15 minutes. Cell apoptosis was then analysed on a flow cytometer (BD Biosciences).

### 2.8 Western blot

Total proteins from cells were extracted by using ice‐cold radioimmunoprecipitation assay lysis buffer (Beyotime), and the protein concentrations were quantified using the Bio‐Rad Protein Assay kit (Thermo Fisher Scientific). Subsequently, equal amounts of protein samples were separated by 10% sodium dodecyl sulphate‐polyacrylamide gel electrophoresis, then transferred to the polyvinylidene fluoride membranes (Millipore). The membranes were then blocked with 5% non‐fat milk for 2 hours at room temperature and incubated with the corresponding primary antibodies against cleaved caspase‐3 (1:1000), cleaved caspase‐9 (1:1000), Bax (1:1500), TGFBR2 (1:1000), phosphorylated Smad3 (p‐Samd3; 1:1000), Smad3 (1:1500) and β‐actin (1:2000; Cell Signaling Technology) overnight at 4°C. After being washed with phosphate‐buffered saline with Tween‐20, the membranes were incubated with the horseradish peroxidase‐conjugated secondary antibodies for 2 hours at room temperature. Signals visualization was conducted by using Enhanced Chemiluminescence Substrate kit (Millipore).

### 2.9 Luciferase reporter assay

The wild‐type luciferase reporter vectors were constructed by cloning the PCR‐amplified fragments of PART‐1 and TGFBR2 3’untranslated region (3’UTR) into the pmirGLO vector (Promega). The mutant fragments of PART‐1 and TGFBR2 3’UTR were amplified and cloned into the pmirGLO vectors to generate the mutant luciferase reporter vectors. For the luciferase reporter assay, cells were co‐transfected with the corresponding reporter vectors and miR‐590‐5p mimic (or miR‐NC) using the Lipofectamine 3000 reagent, and cells were collected for luciferase activity assessment at 48 hours after co‐transfection. The relative luciferase activity was determined by calculated the ratio of Firefly luciferase activity versus Renilla luciferase activity.

### 2.10 RNA immunoprecipitation (RIP)

The immunoprecipitation between PART‐1 and miR‐590‐3p was performed using EZ‐Magna RIP^™^ RNA‐Binding Protein Immunoprecipitation Kit (Millipore) according to previous studies.^11^ Briefly, cells were co‐transfected with pcDNA3.1‐MS2, pcDNA3.1‐PART‐1‐WT‐MS2, or pcDNA3.1‐PART‐1‐MUT‐MS2, and at 48 hours after transfection, cells were processed to perform RIP using EZ‐Magna RIPTM RNA‐Binding Protein Immunoprecipitation Kit according to the manufacturer's protocol.

### 2.11 Statistical analyses

All the data analyses were performed using GraphPad Prism 5.0 (GraphPad Software). All the data were presented as mean ± standard deviation. The comparison between two groups was analysed by Student's *t* test, and the comparison among multiple groups was analysed by one‐way analysis of variance followed by Bonferroni's post hoc test. The correlation between two variables was analysed by Pearson correlation analysis. *P* < .05 was considered statistically significant.

## 3 RESULTS

### 3.1 The expression level of PART‐1 in OA and normal cartilage tissues

**Figure 1:**
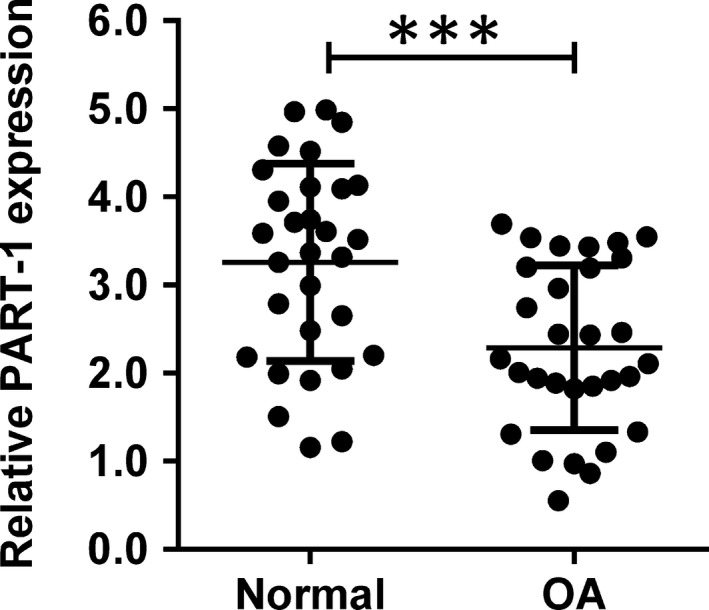
PART‐1 was down‐regulated in OA articular cartilages. The relative expression level of PART‐1 in the clinical samples from normal or OA articular cartilages was analysed by qRT‐PCR. N = 30, significant differences were presented as ***P < .001

Firstly, we detected the expression level of PART‐1 in donated OA and normal cartilage tissues from the patients. As shown in Figure 1, PART‐1 was significantly down‐regulated in the OA cartilage tissues when compared with the normal ones (Figure 1).

### 3.2 The effects of PART‐1 silence/overexpression on the apoptosis of chondrocytes

**Figure 2:**
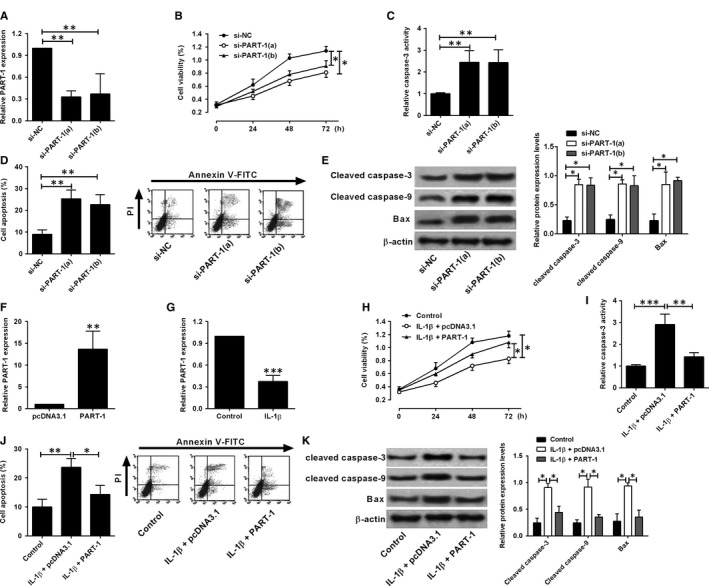
Effects of PART‐1 on the cell viability and apoptosis of chondrocytes. A‐E, Chondrocytes were transfected with PART‐1 siRNAs (si‐PART‐1(a) or (b)) or the scrambled negative controls; at 24 h after transfection, (A) the PART‐1 expression was analysed by qRT‐PCR; at 0, 24, 48 and 72 h after transfection, (B) cell viability was determined by CCK‐8 assay; at 24 h after transfection, (C) caspase‐3 activity was measured by the caspase‐3 activity kit, (D) cell apoptotic rates were analysed by flow cytometry; (E) protein levels were determined by Western blot assay. (F) Cells were transfected with pcDNA3.1‐PART‐1 or pcDNA3.1; at 24 h after transfection, the PART‐1 expression was determined by qRT‐PCR assay. (G) Cells were treated with IL‐1β for 24 h, and the PART‐1 expression was determined by qRT‐PCR assay. H‐K, Cells with IL‐1β treatment were transfected pcDNA3.1 or pcDNA3.1‐PART‐1, and at 0, 24, 48 and 72 h after transfection, (H) cell viability was determined by CCK‐8 assay; at 24 h after transfection, (I) caspase‐3 activity was measured by the caspase‐3 activity kit, (J) cell apoptotic rates were analysed by flow cytometry; (K) protein levels were determined by Western blot assay. N = 3; significant differences were presented as *P < .05, **P < .01 and ***P < .001

The silence of PART‐1 was achieved by transient transfection of specific siRNAs for PART‐1 (si‐PART‐1 (a) or (b)). The PART‐1 expression level was significantly decreased in the chondrocytes with PART‐1 siRNAs transfection (Figure 2A). The cell viability was assessed at the time‐point of 0, 24, 48 and 72 hours after siRNAs transfection. Knock‐down of PART‐1 decreased the cell viability when compared with nonsense siRNA group (Figure 2B). In addition, the caspase‐3 activity and cell apoptotic rates were also increased markedly upon PART‐1 knock‐down (Figure 2C,D). Meanwhile, the pro‐apoptotic proteins including cleaved caspase‐3 and caspase‐9 as well as Bax were up‐regulated in the chondrocytes with PART‐1 knock‐down (Figure 2E). Collectively, silence of PART‐1 promoted chondrocytes apoptosis.

Next, we explored the effects of PART‐1 overexpression on the IL‐1β‐stimulated chondrocytes, as IL‐1β, an inflammatory cytokine, is increased in OA joint tissues and plays an important role in OA development.^13^ Overexpression of PART‐1 was achieved by transfecting chondrocytes with pcDNA3.1‐PART‐1. The PART‐1 expression level was increased to about 12‐fold in chondrocytes after pcDNA3.1‐PART‐1 transfection when compared with the empty vector pcDNA3.1 (Figure 2F). After IL‐1β stimulation, the PART‐1 expression level dramatically decreased (Figure 2G). Moreover, IL‐1β stimulation caused a decrease in cell viability (Figure 2H) and an increase in caspase‐3 activity (Figure 2I) and cell apoptotic rates (Figure 2J). Meanwhile, the cleaved caspase‐3 and caspase‐9 as well as Bax were also up‐regulated upon IL‐1β stimulation (Figure 2K). Overexpression of PART‐1 could reverse the effects induced by IL‐1β stimulation in chondrocytes (Figure 2H‐K).

### 3.3 PART‐1 interacts with miR‐590‐3p

**Figure 3:**
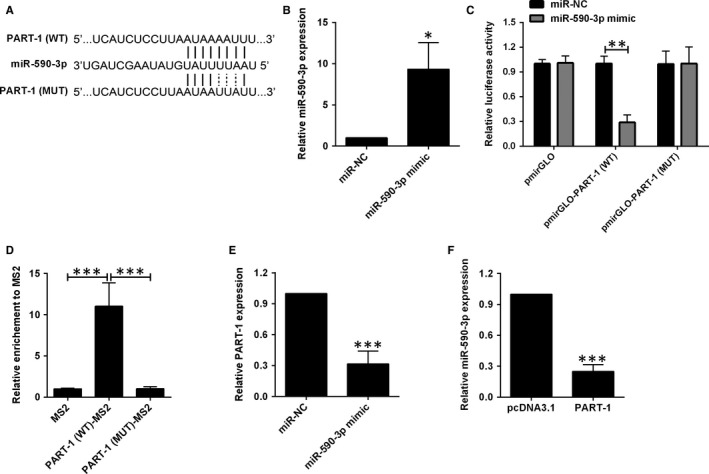
PART‐1 sponged miR‐590‐3p and repressed miR‐590‐3p expression. (A) The interaction between PART‐1 and miR‐590‐3p as predicted by LncBase Predicted V2.0. (B) miR‐590‐3p expression was detected in cells transfected with miR‐590‐3p mimic or miR‐NC. (C) Luciferase activity was determined in cells co‐transfected with different luciferase reporter vectors and miRNAs. (D) RIP analysis of the interaction between PART‐1 and miR‐590‐3p. (E) PART‐1 expression was detected in cells transfected with miR‐590‐3p or miR‐NC. (F) miR‐590‐5p expression was detected in cells transfected with pcDNA3.1 or pcDNA3.1‐PART‐1. N = 3, significant differences were presented as *P < .05, **P < .01 and ***P < .001

It is well known that lncRNAs exert their effects through interacting with miRNAs to regulate downstream targets. Online prediction software indicates that several miRNAs are the potential targets of PART‐1, and miR‐590‐3p was selected as the target for examination, due to the regulatory effects of miR‐590‐3p on apoptosis in different types of cells.^14^, ^15^, ^16^ Consequently, we have constructed two luciferase reporter vectors, one containing wide‐type miR‐590‐3p–binding site and the other containing the mutant one (Figure 3A). Cells were co‐transfected with miR‐590‐3p mimic (or miR‐NC) and the corresponding luciferase reported vectors. The mimic increased the expression level of miR‐590‐3p by around 7‐fold, which was checked prior to luciferase reporter assay (Figure 3B). The luciferase activity was remarkably decreased after transfection with the wide‐type vector and miR‐590‐3p mimic while the luciferase activity was not affected by the mutant vector and miR‐590‐3p mimic transfection (Figure 3C). To validate the direct binding between PART‐1 and miR‐590‐3p, MS2‐RIP was used to pull down the complex of miR‐590‐3p and PART‐1. The data showed that PART‐1 pull down was significantly enriched for miR‐590‐3p (Figure 3D). In addition, transfection with miR‐590‐3p mimic down‐regulated PART‐1 and vice versa (Figure 3E,F).

### 3.4 MiR‐590‐3p targets TGFBR2

**Figure 4:**
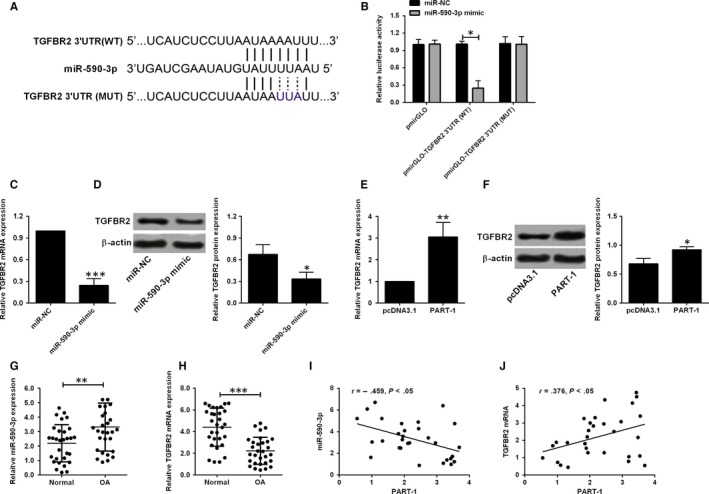
MiR‐590‐3p targeted TGFBR2 and repressed TGFBR2 expression. (A) The interaction between miR‐590‐3p and TGFBR2 3'UTR. (B) Luciferase activity was determined in cells co‐transfected with different luciferase reporter vectors and miRNAs. (C) The mRNA and (D) protein expression levels of TGFBR2 in cells transfected with miR‐590‐3p mimic or miR‐NC. (E) The mRNA and (F) protein expression levels of TGFBR2 in cells transfected with miR‐590‐3p mimic or miR‐NC. (G) MiR‐590‐3p and (H) TGFBR2 mRNA expression in the clinical samples from normal (n = 30) or OA (n = 30) articular cartilages were analysed by qRT‐PCR. (I) The correlation between PART‐1 and miR‐590‐3p expression level in OA articular cartilages. (J) The correlation between PART‐1 and TGFBR‐2 mRNA expression level in OA articular cartilages. N = 3, significant differences were presented as *P < .05, **P < .01 and ***P < .001

Subsequently, we also used online software to predict the potential targets of miR‐590‐3p and identified TGFBR2 as the potential target of miR‐590‐3p. Consequently, two luciferase reporter vectors containing the wide‐type/mutant miR‐590‐3p–binding site for TGFBR2 3’UTR were constructed (Figure 4A). Cells were co‐transfected with miR‐590‐3p mimic and the luciferase reported vectors. MiR‐590‐3p mimic decreased the luciferase activity when transfecting with the wide‐type vector and had no effect on the mutant one (Figure 4B). Moreover, miR‐590‐3p mimic down‐regulated the expression of TGFBR2 in both mRNA and protein levels (Figure 4C,D). On the other hand, overexpression of PART‐1 up‐regulated the TGFBR2 expression in mRNA and protein levels (Figure 4E,F). Next, we checked the clinical samples for the expression profile of miR‐590‐3p and TGFBR2. The expression level of miR‐590‐3p was higher in the OA group than that in the normal group (Figure 4G). The TGFBR2 mRNA level was lower in the OA group than that in the normal group (Figure 4H). The correlation analysis showed miR‐590‐3p was negatively associated with PART‐1 while TGFBR2 was positively associated with PART‐1 (Figure 4G‐J).

### 3.5 PART‐1 promotes cell apoptosis through miR‐590‐3p/TGFBR2 in chondrocytes

**Figure 5:**
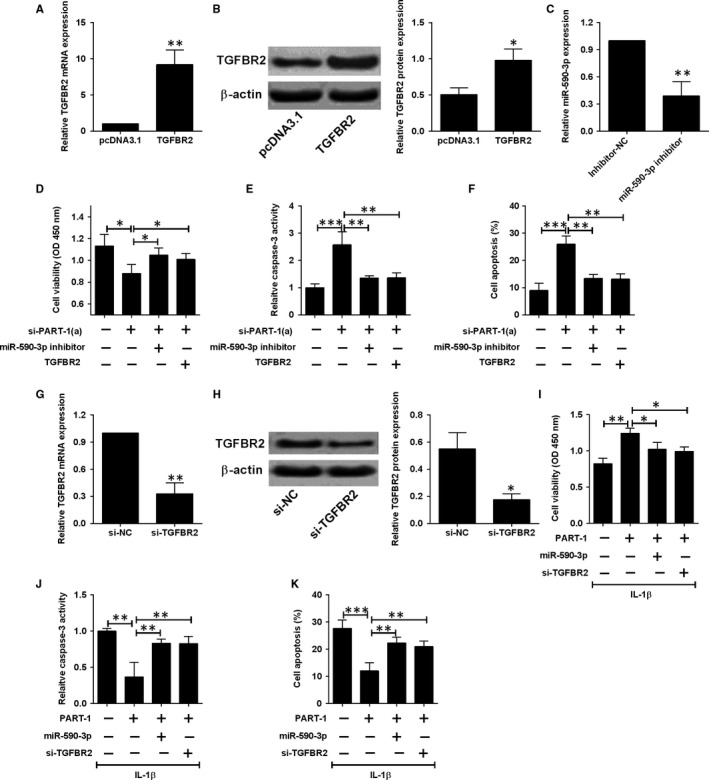
PART‐1 regulated chondrocyte viability and apoptosis via targeting miR‐590‐3p/TGFBR2 axis. (A) The mRNA and (B) protein expression levels of TGFBR2 were detected in cells transfected with pcDNA3.1‐TGFBR2 or pcDNA3.1. (C) MiR‐590‐3p expression in cells transfected with miR‐590‐3p inhibitor or inhibitor‐NC. (D) Cell viability, (E) caspase 3 activity and (F) cell apoptotic rates were detected in cells co‐transfected with si‐PART‐1 (a) + miR‐590‐3p inhibitor, si‐PART‐1 (a) +pcDNA3.1‐TGFBR2, or their respective NCs. (G) The mRNA and (H) protein levels of TGFBR2 were determined in cells transfected with si‐TGFBR2 or si‐NC. (I) Cell viability, (J) caspase 3 activity and (K) cell apoptotic rates were detected in IL‐1β‐treated cells that were co‐transfected with pcDNA3.1‐PART‐1 + miR‐590‐3p mimic, pcDNA3.1‐PART‐1 + si‐TGFBR2, or their respective NCs. N = 3, significant differences were presented as *P < .05, **P < .01 and ***P < .001

Ectopic expression of TGFBR2 was achieved by transfection with pcDNA3.1‐TGFBR2 and dramatic increase in both mRNA and protein levels were observed after pcDNA3.1‐TGFBR2 transfection in chondrocytes (Figure 5A,B). Down‐regulation of miR‐590‐3p was achieved through specific miR‐590‐3p inhibitor transfection in chondrocytes (Figure 5C). For the rescue experiments, down‐regulation PART‐1 decreased the cell viability and increased the caspase‐3 activity and apoptotic rates, which can be partially reversed by miR‐590‐3p silence or TGFBR2 overexpression (Figure 5D‐F). Knock‐down of TGFBR2 was achieved by siRNA transfection and both mRNA and protein levels were remarkably decreased in chondrocytes with TGFBR2 siRNA transfection (Figure 5G,H). Overexpression of PART‐1 increased the cell viability and decreased the caspase‐3 activity and apoptotic rates, and the effects were partially attenuated by miR‐590‐3p overexpression or silence of TGFBR2 in IL‐1β stimulated chondrocytes (Figure 5I‐K).

### 3.6 PART‐1/miR‐590‐3p modulated transforming growth factor‐beta (TGF‐β)/Smad3 pathway

**Figure 6:**
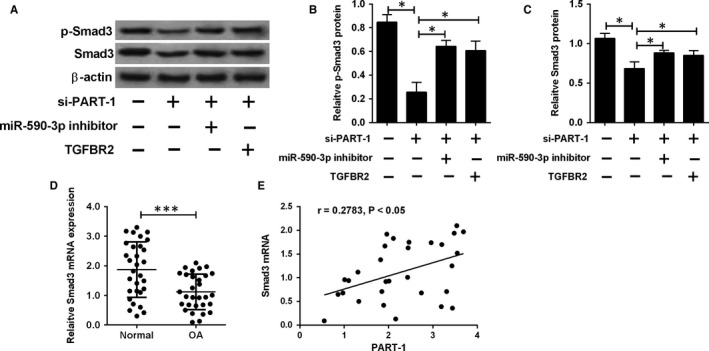
PART‐1 regulated Smad3 expression in chondrocytes. (A‐C) The protein levels of p‐Smad3 and Smad3 were determined in cells co‐transfected with si‐PART‐1 (a) + miR‐590‐3p inhibitor, si‐PART‐1 (a) +pcDNA3.1‐TGFBR2, or their respective NCs. (D) Smad3 mRNA expression in the clinical samples from normal (n = 30) or OA (n = 30) articular cartilages were analysed by qRT‐PCR. (E) The correlation between PART‐1 and Smad3 mRNA expression level in OA articular cartilages. N = 3, significant differences were presented as *P < .05 and ***P < .001

Smad3 protein is a key component of TGF‐β pathway. The following studies explored the modulation of PART‐1/miR‐590‐3p on Smad3. Knock‐down of PART‐1 down‐regulated both Smad3 and p‐Smad3 protein levels, which was reversed through miR‐590‐3p inhibition or TGFBR2 overexpression (Figure 6A‐C). For the clinical samples, Smad3 expression level was lower in the OA group than that in the normal group and was positively associated with the PART‐1 expression level (Figure 6D,E).

## 4 DISCUSSION

OA is a heterogeneous disease affects all synovial joints, such as spine, hips or knees. OA can also be regarded as a degenerative disease as the main pathological features of OA are progressive articular cartilage degeneration.^17^ Ageing is the major risk factor for OA and other factors including obesity and heredity also contribute to the development of OA.^18^ The molecular mechanisms for OA are not fully elucidated, and extracellular matrix destruction, inflammatory responses, or chondrocytes apoptosis are closely involved in the OA progression.^19^ In this study, we have focused on apoptosis and explored the modulation effect of lncRNA PART‐1 on chondrocytes apoptosis.

Reduced chondrocyte number due to apoptosis will cause cartilage degeneration in the development of OA. Previously, some studies focusing on aberrantly expressed lncRNAs in OA revealed that lncRNA growth arrest special 5 induced human chondrocytes apoptosis through down‐regulating miR‐21 levels.^20^ In rabbit condylar chondrocytes model, silence of HOX transcript antisense RNA (HOTAIR) attenuated the apoptosis induced by IL‐1β.^20^ In human chondrocytes, HOTAIR aggravated extracellular matrix degradation and chondrocytes apoptosis through up‐regulation of fucosyltransferase 2.^21^ LncRNA PART‐1, located on chromosome 5, however, was rarely reported. It was first identified in human prostate and can modulate androgen receptor‐regulated gene network and involve in prostate carcinogenesis.^22^ Recent studies regarded it as an oncogene for prostate cancer as its expression was up‐regulated and inhibited cell apoptosis.^23^ In addition, the genome‐wide identification of lncRNAs in human intervertebral disc degeneration indicated that PART‐1‐mediated apoptosis may be related to the changes in the vascular endothelial growth factor A (VEGFA),^12^ as VEGFA is essential for the survival of chondrocytes and suppression of VEGFA promoted the apoptosis of chondrocytes.^24^, ^25^, ^26^ Consequently, we hypotheses that PART‐1 may relate to the chondrocyte apoptosis in OA. In the present study, the PART‐1 expression level was down‐regulated in the OA chondrocytes. In addition, silence of PART‐1 decreased the cell viability and promoted chondrocytes apoptosis. Overexpression of PART‐1 could reverse the effects induced by IL‐1β stimulation, thus slowing down the apoptosis. These data indicate that PART‐1 involves in the regulation of chondrocytes apoptosis in OA.

It is well known that lncRNAs act as sponges for miRNAs in the biological or pathological processes. For PART‐1, miR‐590‐3p was found to be the potential target, and RIP and luciferase activity assays confirmed the binding between PART‐1 and miR‐590‐3p. Moreover, miR‐590‐3p was down‐regulated by PART‐1. Next, we predicted the downstream target for miR‐590‐3p and TGFBR2 was the candidate. In TGF‐β signalling pathway, the TGF‐β ligand binds to TGFBR2, which then phosphorylates the downstream receptors. The activated kinase subsequently phosphorylates Smad3, which then forms a heteromeric complex with Smad4, translocates into the nucleus, and regulates target gene transcription, which is essential for cellular functions such as cellular proliferation, differentiation and apoptosis.^27^ In TGFBR2‐knockout mice and Smad3‐knockout mice, they showed an OA‐like phenotype, reduced type II collagen and severe deficiency in aggrecan protein.^28^ Consistently, in our study, the TGFBR2 and Smad3 expression level were lower in the OA tissues than that in the normal tissue, which were positively correlated with the PART‐1 expression level.

In the aspects of clinical/in vivo significance for PART‐1 in OA, some issues are required for consideration in the future studies. The clinical samples examined in this study were limited to the small sample size, thus, more samples from multiple clinical centres should be collected to verify the role of PART‐1 in OA development. Moreover, the immunohistochemistry should be performed in the clinical samples to determine the localization and expression of PART‐1 and its downstream targets in OA cartilage, which may further reveal the clinical significance of PART‐1. In the in vivo side, the animal OA model may be established to study the therapeutic effects of PART‐1 in OA, which will be a focus in our future investigations.

Collectively, the study revealed that lncRNA PART‐1 regulated the apoptosis of chondrocytes in OA by acting as a sponge for miR‐590‐3p, which subsequently regulates TGFBR2/Smad3 signalling. PART‐1 has the potential to be a therapeutic target or biomarker for OA.

## CONFLICT OF INTEREST

None.

## AUTHOR CONTRIBUTIONS

CL and KP designed and performed the experiments; ZL and SH performed the data analysis; YC revised the language of the manuscript; KP wrote the manuscript; all the authors approved the manuscript for submission.

## Data Availability

All the data generated in this study are available upon request.
